# Enhancing the thermoelectric figure of merit in engineered graphene nanoribbons

**DOI:** 10.3762/bjnano.6.119

**Published:** 2015-05-18

**Authors:** Hatef Sadeghi, Sara Sangtarash, Colin J Lambert

**Affiliations:** 1 Quantum Technology Centre, Department of Physics, Lancaster University, LA1 4YB Lancaster, UK

**Keywords:** graphene nanoribbons, quantum transport, thermal conductance, thermoelectric figure of merit, thermopower

## Abstract

We demonstrate that thermoelectric properties of graphene nanoribbons can be dramatically improved by introducing nanopores. In monolayer graphene, this increases the electronic thermoelectric figure of merit *ZT*_e_ from 0.01 to 0.5. The largest values of *ZT*_e_ are found when a nanopore is introduced into bilayer graphene, such that the current flows from one layer to the other via the inner surface of the pore, for which values as high as *ZT*_e_ = 2.45 are obtained. All thermoelectric properties can be further enhanced by tuning the Fermi energy of the leads.

## Introduction

Nowadays, the performance of nanoelectronic devices is limited by dissipated power rather than available clock speeds [1]. To address this issue, thermoelectric energy conversion may be an essential ingredient in the design of the next generation of integrated electronics, optoelectronic and photonic devices [2]. On the one hand efficient thermoelectricity requires a strongly suppressed thermal conductivity (κ) since the performance of thermoelectric devices is inversely proportional to the thermal conductivity. On the other hand, the cooling of local hot spots requires a high thermal conductivity [3]. Thermal conductance in a solid is defined by Fourier’s law, 
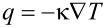
 where *q* is the heat flux, κ = κ_pl_ + κ_e_ is the thermal conductance due to phonons (κ_pl_) and electrons (κ_e_) and 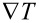 is the temperature gradient [1]. Nanostructures show significantly different thermal properties than bulk crystals in which acoustic phonons are the main heat carriers. The reasons for this are changes in the phonon density of states, an increased phonon-boundary scattering and the dispersion of the nanostructures in low dimensional semiconductors [2,4–6].

The efficiency of thermoelectric materials and devices is determined by their thermoelectric figure of merit (*ZT* = *S*^2^*GT*/κ) where *S* is the Seebeck coefficient, which depends on the asymmetry of the density of states around the Fermi level, *G* is the electrical conductance and *T* is the temperature [7]. Similarly, the electronic thermoelectric figure of merit also is defined as *ZT*_e_ = *S*^2^*GT*/κ_e_. Since the efficiency of a thermoelectric device can be enhanced by increasing the power factor (*S^2^GT*) or by decreasing the thermal conductance, there is a need to simultaneously increase the Seebeck coefficient and electrical conductance, while reducing in thermal conductance. Since these factors are correlated, increasing *ZT* to values greater than unity is challenging. The most common material used in thermoelectric applications is bismuth and its alloys, which are toxic, expensive and of limited availability. To improve *ZT* in new materials, one promising route has been to take advantage of the reduced phonon thermal conductance (κ_pl_) in low dimensional materials [8]. In what follows we apply this approach to engineered graphene nanoribbons [9–10] and show that introducing nanopores into bilayer graphene [11], a room-temperature *ZT*_e_ higher than 2 could be achieved.

## Computational methods

The electrical conductance *G*(*T*), the electronic contribution to the thermal conductance κ(*T*), the thermopower (Seebeck coefficient) *S*(*T*) and the Peltier coefficient Π(*T*) of a junction as a function of the temperature *T* can be obtained by calculating the transmission probability *T*(*E*) of the electrons with energy *E* passing from one electrode to another. From *T*(*E*), in the linear response the quantity *L_n_*(*T*) is defined as:

[1]
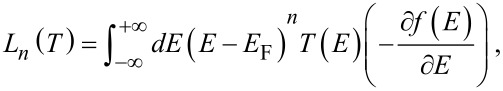


where *f*(*E*) is the Fermi–Dirac probability distribution function (*f*(*E*) = (1 + exp((*E* − *E*_F_)/*k*_B_*T*))^−1^), *T* is the temperature, *e* is electron charge, *h* is Planck’s constant and *E*_F_ is the Fermi energy.

The electrical conductance *G*(*T*) as a function of the temperature *T* is then given by the Landauer formula *G*(*T*) = *G*_0_*L*_0_(*T*), where *G*_0_ = 2*e*^2^/*h* is the conductance quantum. The electronic thermal conductance κ(*T*), the Seebeck *S*(*T*) and Peltier Π(*T*) coefficients are also given by [12]:

[2]
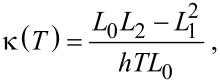


[3]
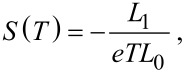


[4]
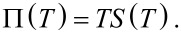


To find the optimized geometry and ground state Hamiltonian of the structure analogously as described in [9], we employed the SIESTA [13] implementation of DFT using the generalized gradient approximation (GGA) of the exchange and correlation functional with the Perdew–Burke–Ernzerhof parameterization (PBE) [14] a double zeta polarized basis set, a real-space grid defined with a plane wave cut-off energy of 250 Ry and a maximum force tolerance of 40 meV/Å. From the converged DFT calculation, the underlying mean-field Hamiltonian was combined with the GOLLUM [12] implementation of the non-equilibrium Greens function (NEGF) method. This yields the transmission coefficient *T*(*E*) for electrons of energy *E* (passing from the source to the drain) via the relation [15]

[5]
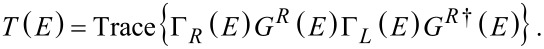


In this expression, Γ_L,R_(*E*) = *i*(Σ_L,R_(*E*) − Σ_L,R_^†^(*E*)) describe the level broadening due to the coupling between left (L) and right (R) electrodes and the central scattering region (S) associated with the pore. 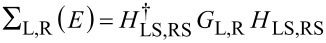 are the retarded self-energies associated with this coupling. *H*_LS,RS_ and *G*_L,R_ are the coupling matrix between LS and RS and the surface Green’s function of the electrodes, respectively. *G^R^* = (*ES* – *H*_S_ – Σ_L_ – Σ_R_)^−1^ is the retarded Green’s function, where *H*_S_ is the Hamiltonian of the scattering region and *S* is the overlap matrix.

## Results and Discussion

### Thermal properties of graphene

Carbon-based materials show a wide range of thermal properties from about 0.01 W·mK^−1^ in amorphous carbon to above 2,000 W·mK^−1^ at room temperature in graphene [1,16–19] and even higher in few layer graphene [20]. This means that 2D graphene and its multilayer counterparts are useful for thermal management applications [21]. The high thermal conductivity of the graphene is mainly due to the high phonon contribution to heat transport. Therefore, for thermoelectricity applications, one needs to engineer phonon transport to achieve a low thermal conductivity. Moreover, graphene is a zero-gap material and not suitable to use as thermoelectric material because of its very small Seebeck coefficient. However, theoretical studies revealed that phonon transport is sensitive to defects, strain, sample size and geometry [21] and it is known that by patterning graphene to form nanoribbons or anti-dots one can suppress the phonon contribution to heat transport [3]. This suppression is supported by experimental data, as reviewed in [2].

Phonon transport in graphene ribbons is limited by the ribbon size and edge characteristics [20]. In addition, equilibrium molecular dynamic simulations showed that hydrogen passivation of the graphene-nanoribbon edges reduces significantly the thermal conductivity [22–23]. Anti-dots in graphene, one can further reduce the phonon thermal conductivity [8]. For example, anti-dots created by removing 2% of the total number of atoms in pristine graphene, reduced the phonon-induced thermal conductivity by almost 50% [21]. However, the stability of anti-dots in graphene is an issue due to self-healing properties of the monolayer graphene [24].

**Figure 1:**
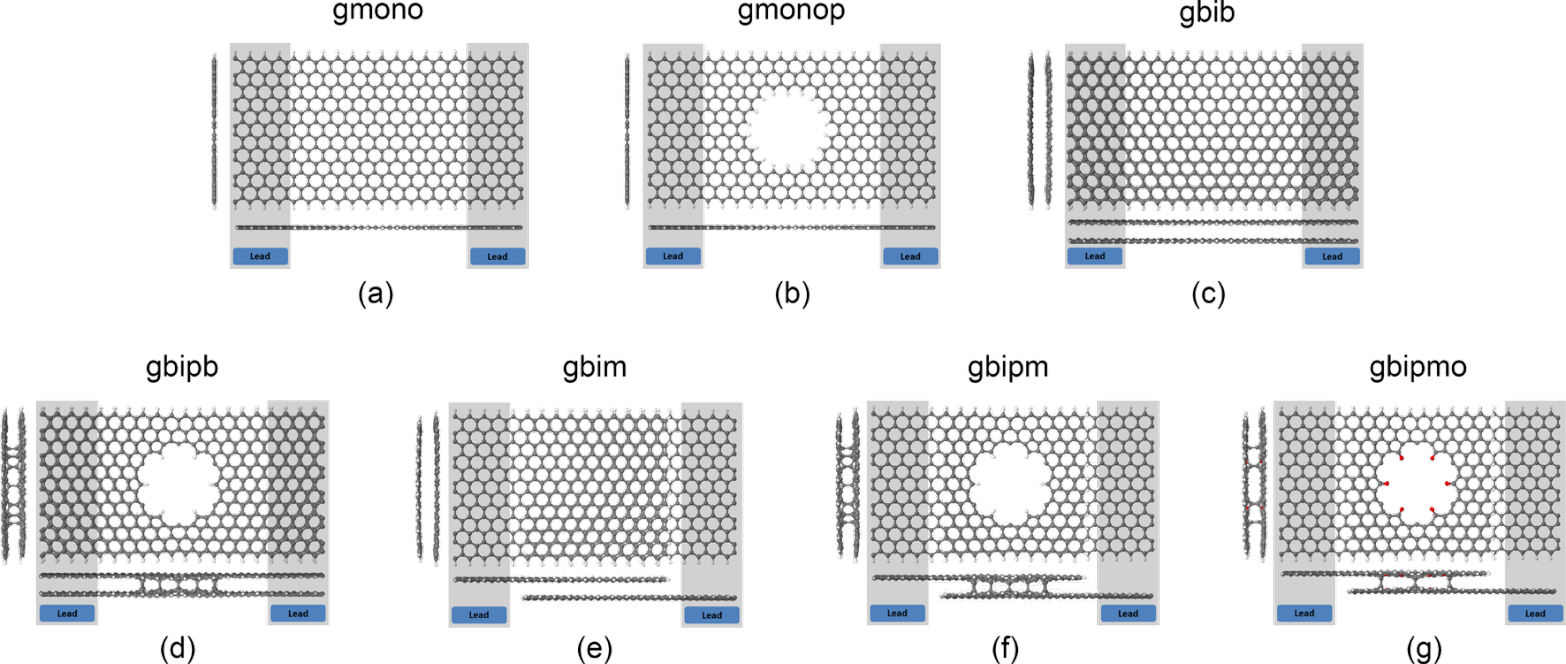
Geometry of the graphene-based structures, (a) monolayer graphene ribbon, (b) monolayer graphene nanopore, (c) AA-bilayer graphene ribbon, (d) engineered bilayer graphene nanopore, (e) AA-bilayer graphene with monolayer lead, (f) engineered bilayer graphene nanopore with monolayer lead and hydrogen termination inside the pore, (g) engineered bilayer graphene nanopore with monolayer lead and oxygen termination inside the pore.

Here, we build upon these results by investigating the thermoelectric properties of various forms of engineered graphene, obtained by sculpting nanopores in bilayer graphene and allowing the pore surface to reconstruct [9]. Pores in bilayer graphene are not only more stable than anti-dots in monolayer graphene, but should also be effective in reducing the phonon contribution to thermal conductance. In what follows, we explore the electrical conductance, thermal conductance, and Seebeck and Peltier coefficients of the range of structures shown in Figure 1. These engineered graphene ribbons include: a zigzag monolayer graphene nanoribbon with hydrogen terminated edges (Figure 1a), a monolayer graphene nanopore with hydrogen terminated edges (Figure 1b), an AA-bilayer graphene nanoribbon (Figure 1c), an engineered bilayer graphene nanopore (Figure 1d), an AA-bilayer graphene with monolayer lead, in which the transport takes place from the top layer to the bottom layer (Figure 1e), an engineered bilayer graphene nanopore with monolayer leads and either hydrogen termination [9] (Figure 1f) or oxygen termination (Figure 1g) inside the pore. The ribbon lengths (*L*) and widths (*W*) in all cases are almost equal (*L* ≈ 6 nm, *W* ≈ 3 nm) and the pores sizes are about 1.3 nm.

### Thermoelectric properties of a monolayer graphene nanoribbon and nanopores

**Figure 2:**
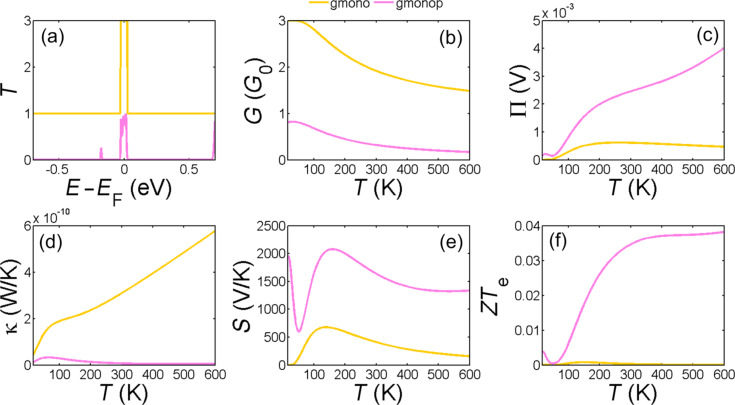
(a) Transmission coefficient *T*(*E*); (b,c) electrical and thermal conductance (*G*, κ), (d,e) Peltier (П) and Seebeck (*S*) coefficients and (f) figure of merit as a function of the temperature in zigzag monolayer graphene nanoribbon (gmono) and monolayer graphene nanopore (gmonop).

Figure 2a shows the transmission coefficient *T*(*E*) for electrons with energies of [−0.7,0.7] eV transmitting from one side of the monolayer graphene nanoribbon and/or monolayer graphene nanopore to the other side. For a perfect crystalline zigzag-edge monolayer graphene nanoribbon with hydrogen-terminated edges (gmono, Figure 1a), *T*(*E*) = 1 outside the Fermi energy and *T*(*E*) = 3 near the Fermi energy. The high *T*(*E*) near the Fermi energy is due to the edge states and band bending, as predicted theoretically [25] and observed experimentally [26–29].

By drilling a hole in the ribbon to create a nanopore as shown in Figure 1b, *T*(*E*) is modified to that shown in Figure 2a (gmonop, pink curve). In this case, the probability of transmitting electrons with energies above or below the Fermi energy is suppressed due to the presence of the pore, whereas the high-transmission feature in the vicinity of the Fermi energy still preserved. This improves the thermopower (Figure 2e) by a factor of 4 and reduces the electronic thermal conductance significantly (Figure 2d), leading to a significant enhancement of *ZT*_e_. However, *ZT*_e_ does not exceed 0.04 at room temperature which is not promising. This agrees with the results reported elsewhere [7].

### Thermoelectric properties of engineered bilayer graphene

**Figure 3:**
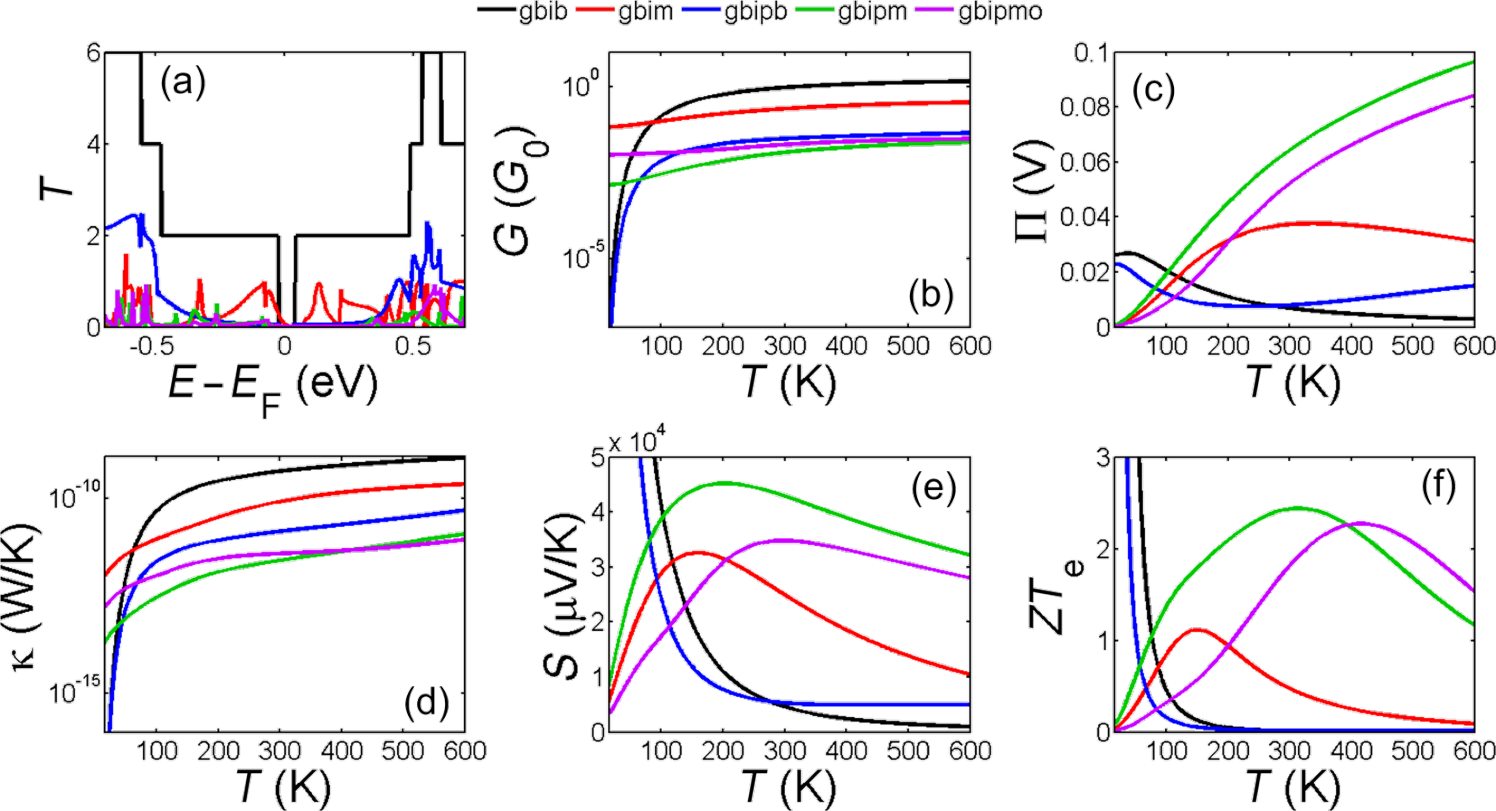
(a) Transmission coefficient *T*(*E*); (b) electrical conductance (*G*), (c) Peltier (П) coefficient, (d) thermal conductance (κ), (e) Seebeck (*S*) coefficient and (f) figure of merit as a function of temperature in zigzag bilayer graphene nanoribbon (gbib), bilayer graphene with monolayer lead (gbim), engineered bilayer graphene nanopore (gbipb), engineered bilayer graphene nanopore with monolayer lead and hydrogen termination in pore side (gbipm), engineered bilayer graphene nanopore with monolayer lead and oxygen termination in pore side (gbipmo).

Figure 3a shows *T*(*E*) for the structures shown in Figure 1c–g. The bilayer graphene nanoribbons with hydrogen-terminated edges have the highest thermal conductance and lowest thermopower amongst all the examples of bilayer graphene. By connecting only the top layer of the left hand side to the left electrode and bottom layer of the right hand side to the right electrode (Figure 1f) so that the current flows through the surface of the pore coupling the top and bottom layers of the bilayer, the thermal conductivity is supressed and *ZT*_e_ is improved but only at low temperatures.

By placing a hole in bilayer graphene and allowing it to be reconstructed, such that the pore edges couple the top and bottom layers, we find that the thermal conductance is significantly suppressed (Figure 3d and Figure 3e). This is even more pronounced for the bilayer nanopore with monolayer leads and hydrogen or oxygen terminations at the inner side of the pore (Figure 1f,g). As shown in Figure 3f, for both hydrogen and oxygen terminations, the high thermopower and low thermal conductance of this engineered bilayer graphene induces a significant increase in the room-temperature figure of merit (*ZT*_e_ ≈ 2.5).

**Figure 4:**
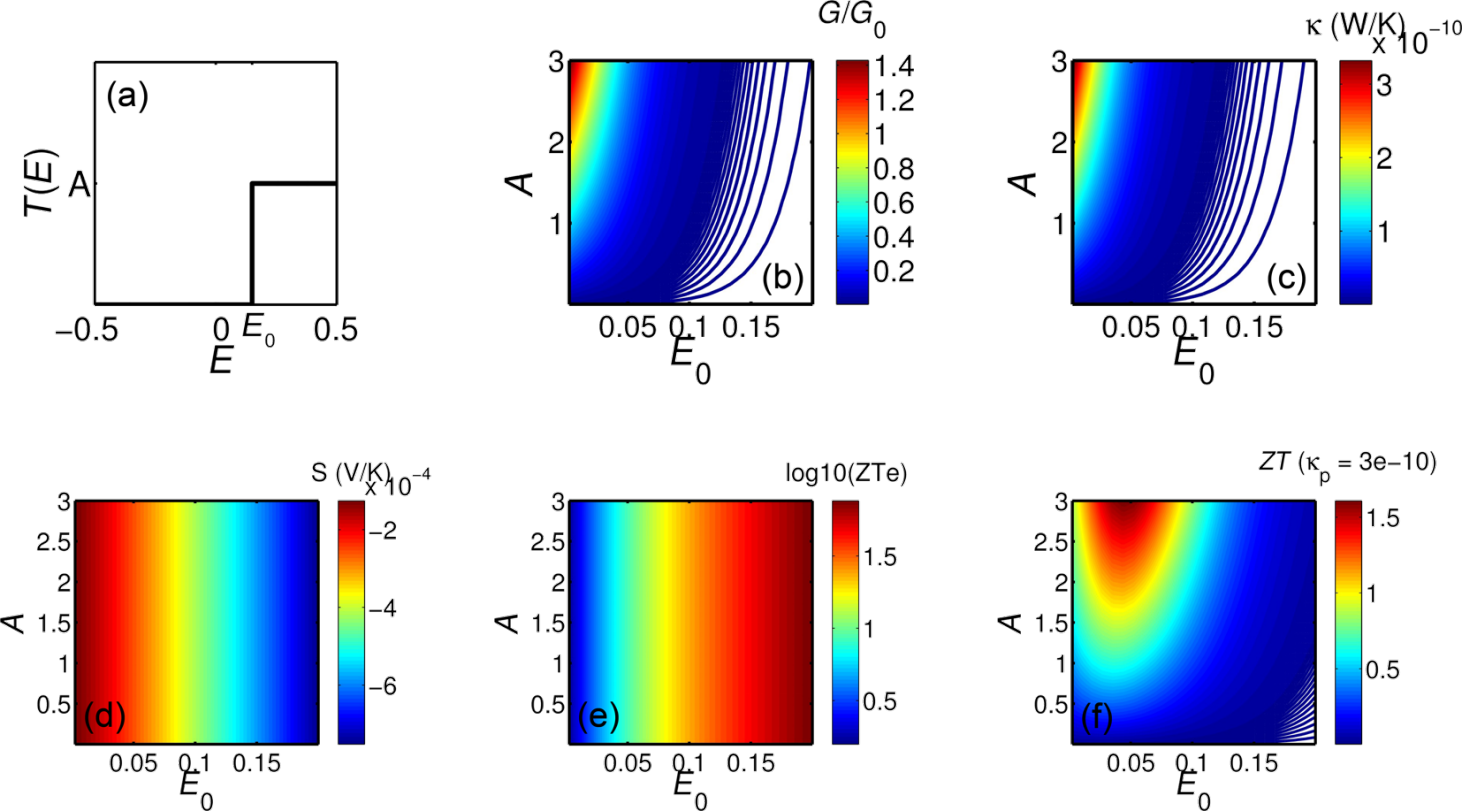
(a) Ideal step function like transmission coefficient *T*(*E*) asymmetric around Fermi energy (*E* = 0), (b) electrical conductance (*G*), (c) thermal conductance (κ), (d) Seebeck coefficient (*S*) and (e) electronic and (f) full figure of merit as a function of position of step function *E*_0_ and the amplitude of *T*(*E*).

To provide insight into the above improvements in *ZT*_e_, we note that an asymmetric delta-function-like peak in the transmission coefficient around the Fermi energy is known to have high *ZT*_e_ [4]. Here we show that the asymmetric step-function-like transmission coefficient *T*(*E*) could lead to high *ZT*_e_. Figure 4a shows the model of an ideal transmission coefficient in the form of a step function near *E*_F_. Figure 4b–e shows the corresponding values of electrical conductance (*G*), thermal conductance (κ), Seebeck (*S*) coefficient and electronic figure of merit as a function of the position of the step function *E*_0_ and the amplitude *A*. It is apparent from Figure 4e that by optimizing the location of the step *E*_0_ one could achieve a high *ZT*_e_. By choosing the phononic contribution to the thermal conductance to be about 5 times higher than the electronic contribution, Figure 4f shows the full *ZT*.

For the structures in Figure 3, *T*(*E*) exhibits gaps rather than step functions near *E*_F_. However, when these are placed asymmetrically relative to *E*_F_, one step-edge of the gap dominates. This gap also needs to be asymmetric around the Fermi energy to deliver high thermopower. By introducing a nanopore in the bilayer graphene (gbipm, gbipb or gbipmo) or considering the transport in the vertical direction (gbim), this gap is obtained. Although transport in the vertical direction (gbim) increases the gap and makes it slightly asymmetrical, the transmission steps are not large enough and or sufficiently asymmetric to overcome thermal broadening at higher temperatures, although such features do improve *ZT*_e_ in low temperatures. Introducing a pore in bilayer graphene with bilayer leads makes the gap too big and step is too far from the Fermi energy and therefore it leads to low *ZT*_e_. However, for gbipm and gbipmo, much better optimization is achieved leading to high *ZT*_e_. This shows how one could engineer the gap size and Fermi energy of a graphene based structure by simply mechanically engineering the bilayer graphene.

**Figure 5:**
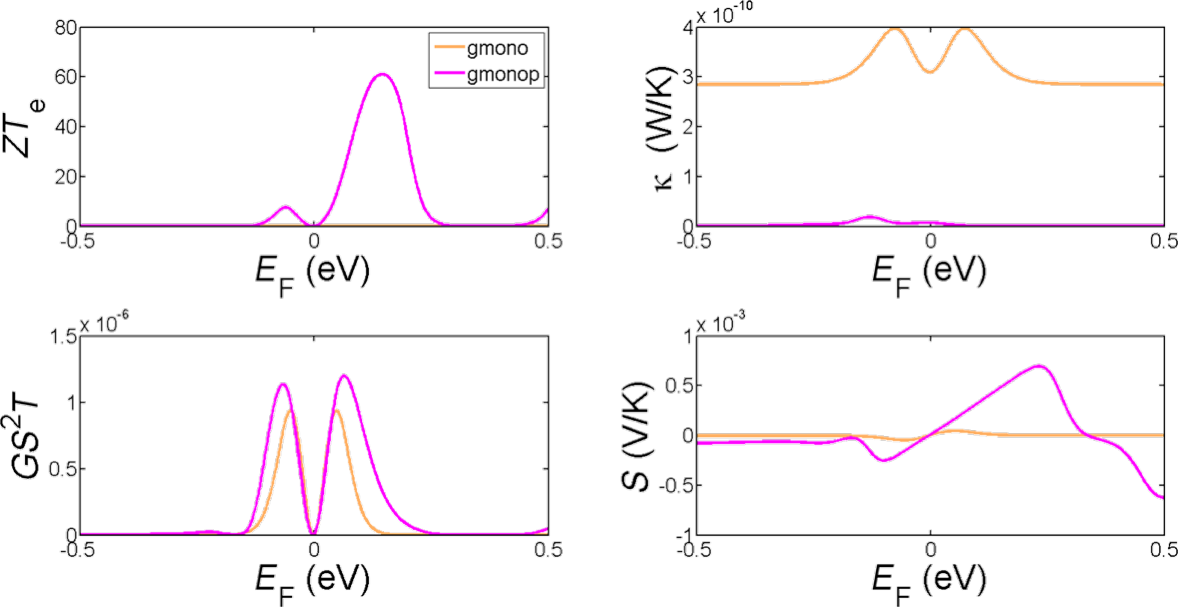
The variation of room-temperature values of *ZT*_e_, *GS*^2^*T*, *S* and κ as a function of the Fermi energy *E*_F_ for a zigzag monolayer graphene ribbon (gmono) and a monolayer graphene nanopore (gmonop).

**Figure 6:**
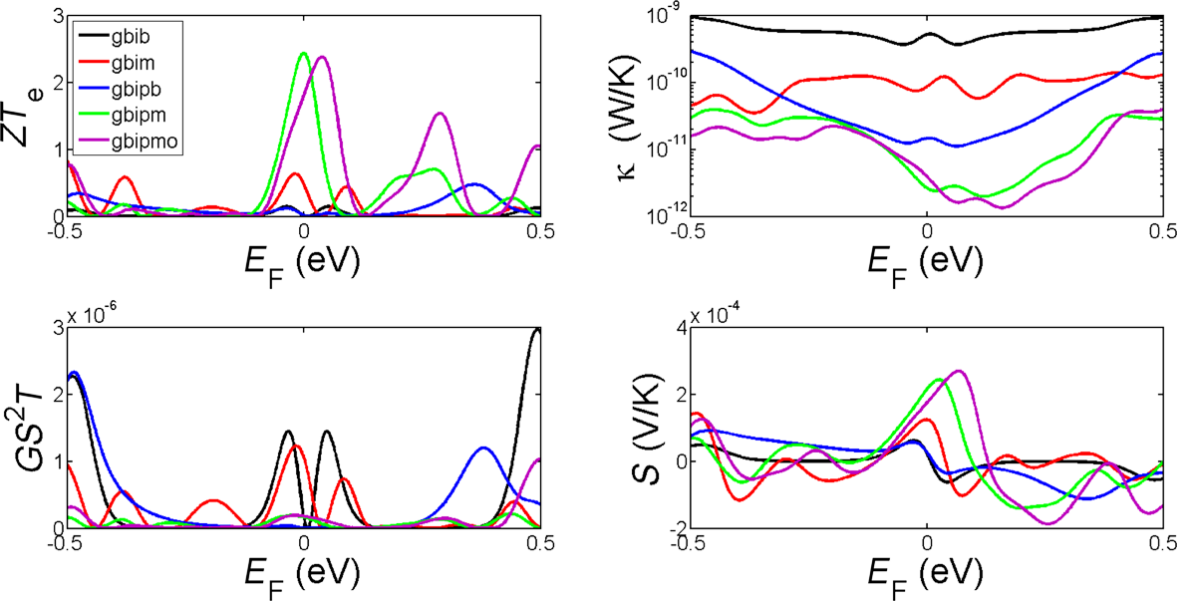
The variation of room-temperature values of *ZT*_e_, *GS*^2^*T, S* and κ as a function of *E*_F_ for zigzag bilayer graphene nanoribbon (gbib), bilayer graphene with monolayer lead (gbim), engineered bilayer graphene nanopore (gbipb), engineered bilayer graphene nanopore with monolayer lead and hydrogen termination in pore side (gbipm), engineered bilayer graphene nanopore with monolayer lead and oxygen termination in pore side (gbipmo).

To further optimise the room-temperature thermoelectric properties of these structures, we now consider the effect of the tuning the Fermi energy. Figure 5 and Figure 6 show the dependence on the Fermi energy of the room-temperature thermoelectric figure of merit *ZT*_e_, the power factor *GS^2^T,* the thermal conductance *κ* and the Seebeck coefficient *S* of the structures shown in Figure 1. These demonstrate that by drilling a pore in both monolayer and bilayer graphene and tuning the Fermi energy, *ZT*_e_ is significantly improved. This improvement is much higher in monolayer graphene as shown in Figure 5 specifically at higher Fermi energies in the range of 0.1–0.2 eV, where the *ZT*_e_ improves by a factor of up to 60.

## Conclusion

We have demonstrated two strategies for increasing *ZT*_e_ in bilayer graphene. First, by connecting the top graphene layer to a cold electrode and the bottom graphene layer to a hot electrode (Figure 1e)*,* not only will the phonon contribution in thermal conductance be reduced due to the fact that the inter-layer coupling is weaker than the intra-layer C–C coupling, but *ZT*_e_ is increased by shifting the Fermi energy to the right (as in p doping) as it is clear by comparing the red and black curves in Figure 6. This improves *ZT*_e_ from 0.01 to 0.5 in *E*_F_ = 0. The second strategy involves introducing pores in bilayer graphene. This shift and improvement of *ZT*_e_ is even higher when a pore is created in both layers, such that the top graphene layer is connected to the bottom graphene layer by the internal surface of the pore, as shown by the green and purple curves in Figure 6. This type of nanostructuring would also reduce the phonon contribution to the thermal conductance. By this technique the Fermi energy is shifted more to the left and *ZT*_e_ increases to 2.45 in the structure shown in Figure 1f. Oxygen or hydrogen termination (Figure 1e and Figure 1f) has a smaller effect in the *ZT*_e_ as shown in Figure 5 (green and purple curves). It is interesting to note that all bilayer structures possess a high thermopower in the range of hundreds of microvolts. Finally, Figure 5 and Figure 6 show that all thermoelectric properties can be further enhanced by tuning the Fermi energy of the leads.
